# Tailoring PLA-Based Composite Membranes with Ionic Liquids for Efficient H_2_/CO_2_ Separation in Reforming Processes

**DOI:** 10.3390/ma19122567

**Published:** 2026-06-13

**Authors:** Dionysios Vroulias, Athina Nikolopoulou, Theophilos Ioannides, Vassilios Dracopoulos

**Affiliations:** Institute of Chemical Engineering Sciences (ICE-HT), Foundation for Research and Technology—Hellas (FORTH), Stadiou St., GR 265 04 Patras, Greecetheo@iceht.forth.gr (T.I.); indy@iceht.forth.gr (V.D.)

**Keywords:** polylactic acid, ionic liquids, gas separation, H_2_/CO_2_ selectivity, reforming

## Abstract

Hydrogen (H_2_), produced from syngas and the Water–Gas Shift reaction, plays a vital role as both an energy carrier and an essential industrial feedstock. This preliminary study examines the effect of incorporating ionic liquids into PLA membranes for the separation of hydrogen (H_2_) from carbon dioxide (CO_2_), aiming to provide a more energy-efficient alternative to the conventional Pressure Swing Adsorption process. Specifically, neat PLA and composite membranes containing cholinium-based ionic liquids at concentrations of 3% and 10% were fabricated. Their thermal properties and microstructural characteristics were systematically analyzed, alongside their gas separation performance. The most promising membrane was further evaluated under humid conditions to assess the impact of water presence. The PLA membrane incorporating 3% cholinium glycinate ionic liquid demonstrated the best performance, achieving a hydrogen permeability of 111 Barrer and an H_2_/CO_2_ selectivity of 8.2, surpassing the Robeson Upper Bound reported in 2008. However, the presence of water led to a decline in separation performance, indicating that effective water removal is necessary prior to membrane application in hydrogen purification.

## 1. Introduction

Hydrogen (H_2_) is currently one of the most important feedstock gases in the chemical industry, playing a crucial role in chemical processes such as ammonia and methanol production, petroleum refining, and hydrodesulfurization [1]. In addition to its industrial applications, H_2_ is increasingly viewed as a promising, environmentally friendly energy carrier for the future [2]. However, before H_2_ can be used, its purification must be adequately addressed.

In the petrochemical industry, H_2_ is produced both as a byproduct of industrial processes such as naphtha reforming and cracking, and through large-scale syngas production methods. The main industrial route is the steam reforming of hydrocarbons (Equation (1)), which generates a mixture of H_2_, carbon monoxide (CO), carbon dioxide (CO_2_), and water (H_2_O), while CO_2_ is also formed via the Water–Gas Shift (WGS) reaction (Equation (2)) [3]. Additionally, partial oxidation (Equation (3)) and autothermal reforming (Equation (4)) can contribute to H_2_ production under controlled conditions [3].

(1) $$\mathrm{Steam}\;\mathrm{Reforming}:\;2C_{n}H_{m}+2nH_{2}O\to 2nCO+\left(2n+m\right)H_{2}$$

(2) $$\mathrm{Water}–\mathrm{Gas}\;\mathrm{Shift}:\;CO+H_{2}O\to CO_{2}+H_{2}$$

(3) $$\mathrm{Partial}\;\mathrm{Oxidation}:\;{2C}_{n}H_{m}+nO_{2}\to 2nCO+mH_{2}$$

(4) $$\mathrm{Autothermal}\;\mathrm{Reforming}:\;{4C}_{n}H_{m}+2nH_{2}O+nO_{2}\to 4nCO+2\left(n+m\right)H_{2}$$

Subsequently, CO is converted to CO_2_ via a two-stage Water–Gas Shift reaction carried out in high- and low-temperature reactors and trace amounts of residual CO can be eliminated by absorption in Cu(I)-based solutions [4]. The resulting CO_2_ is then separated from H_2_ using Pressure Swing Adsorption units equipped with selective adsorbents, such as calcium oxide (CaO), or captured in monoethanolamine (MEA) solutions [5]. These combined purification steps ultimately yield a high-purity H_2_ stream.

A key disadvantage of the above purification methods is their high energy consumption due to the regeneration of the liquid or solid sorbent in absorption and adsorption processes, respectively. Membrane technology offers a promising alternative to these conventional methods as it can operate at ambient temperature without any phase change, resulting in significant energy savings and reduced operating costs. Additional benefits include simple operation, facile maintenance and small footprint [6].

Regarding the type of material, there are three main categories of H_2_-selective membranes: metallic (Pd, Pt, etc.) [7], inorganic (silica, carbon and zeolite) and polymeric membranes. Although metallic membranes are highly permeable and selective membranes towards H_2_, the expense and their risk of processability are a big challenge to be used in large-scale manufacturing. These challenges also apply to zeolite membranes, with mechanical fragility, difficulties in scale-up, fouling, and pore blockage representing additional concerns.

The current market is dominated by polymeric membranes due to their excellent thin-film forming ability, strong chemical, thermal and mechanical properties, and attractive separation performance. However, many polymeric membranes exhibit an inherent trade-off behavior between permeability and selectivity, whereby highly permeable membranes tend to have low selectivity, and vice versa [8]. For this reason, only a small fraction of polymers is suitable for practical commercial H_2_ separation from CO_2_ [9]. Membranes showing high H_2_ permeation flux combined with good H_2_ selectivity and high mechanical resistance are desirable.

Among emerging alternatives, polylactic acid (PLA) has attracted increasing attention as a relatively new material in membrane technology. Conventional PLA membranes exhibit several limitations in gas separation applications, including low gas permeability and insufficient selectivity for key gas pairs such as H_2_/CO_2_. Only recently has Iulianelli et al. prepared a PLA membrane (26 μm thick) by phase inversion technique, which exhibited an H_2_ permeability of 25 Barrer with an ideal selectivity H_2_/CO_2_ around 25, thus surpassing Robeson’s upper bound (2008) [10]. PLA membranes are also sensitive to environmental factors, particularly humidity, which can compromise their structural integrity and transport behavior. Furthermore, their inherent brittleness and restricted ability to tune free volume further limit their overall separation efficiency.

Ionic liquids (ILs) can help PLA membranes mainly by modifying polymer structure and introducing more favorable gas transport pathways. ILs are an emerging class of chemical compounds with significant potential as plasticizers, thereby improving membrane flexibility, durability, and permeability characteristics. In addition, they exhibit negligible volatility, making them attractive as environmentally friendly solvents [11]. They are also non-flammable, exhibit relatively high thermal stability, maintain stability in the presence of water, and can be readily regenerated [11]. Among them, cholinium-based ILs are particularly appealing due to their low toxicity and enhanced environmental compatibility. Their incorporation into PLA is therefore consistent with current efforts to develop membrane materials based on renewable and environmentally benign components. So far, several studies have investigated the effect of ILs on PLA matrices [12,13,14], focusing on their physicochemical characteristics, while IL/PLA systems have primarily been explored for food packaging and biomedical and pharmaceutical applications.

The novelty of this work lies in the exploration of cholinium-based ILs as additives in PLA membranes for H_2_/CO_2_ separation, a topic that has received very limited attention in the literature. This work presents a preliminary investigation into the effect of cholinium-based ILs on H_2_/CO_2_ separation performance when combined with PLA matrices. Their thermal properties and microstructural characteristics were studied, alongside their gas separation performance. The most promising membrane was further evaluated under humid conditions to assess the impact of water presence.

## 2. Materials and Methods

### 2.1. Materials

Glycine (≥99%), L-lysine (≥98%) and choline hydroxide solution (46 wt.% in H_2_O) were supplied by Sigma-Aldrich (Burlington, MA, USA). Acetone (Multisolvent HPLC grade ACS ISO UV-VIS) and polylactic acid (PLA, 4060) were purchased from Scharlab│The Lab Sourcing Group (Barcelona, Spain) and NatureWorks (Plymouth, MN, USA), respectively. All gases (CO_2_, CH_4_, H_2_, N_2_ and He) with a purity of 99.999% were provided by Linde Hellas (Athens, Greece).

### 2.2. IL Preparation

All ILs were synthesized via an acid–base neutralization reaction. For the preparation of cholinium acetate ([Ch][Ac]) IL, 16.8 mL (16.8 mmol) of aqueous acetic acid solution 1 M were mixed with 4.13 mL (16.8 mmol) of aqueous choline hydroxide 46 wt.% at RT. In the case of cholinium glycinate ([Ch][Gly]) or cholinium lysinate ([Ch][Lys]) ILs, 1.2612 g (16.8 mmol) of glycine or 2.4560 g (16.8 mmol) of lysine were used, respectively. After stirring for 30 min, water evaporated at 50 °C under vacuum, and the resulting viscous products were dried at 50 °C under vacuum for 48 h. The yield of the IL preparation was quantitative (100%) for all synthesized ILs.

### 2.3. Membrane Fabrication

**Scheme 1:**
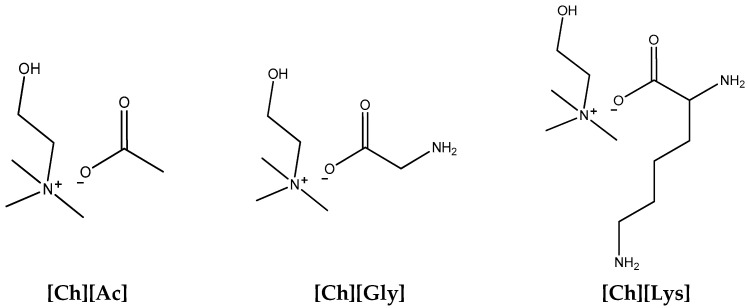
Molecular structures of ILs employed in the fabrication of IL/PLA composite membranes.

PLA and IL/PLA membranes were prepared using the casting method. First, 1.2500 g of PLA were dissolved in 25 mL of acetone at RT. Either 0 mg, 1.5 mg, 4.6 mg, 7.9 mg or 16.7 mg of each IL was added to 3 mL of the polymer solution under stirring and then the mixture was poured onto a flat Teflon plate. Afterwards, the solvent was left to evaporate at RT for 1 h and, hence, under vacuum at 40 °C overnight. The membranes synthesized are symbolized as x%IL/PLA, where x denotes the wt.% concentration of IL used, as shown in Scheme 1.

### 2.4. Material Characterization

^1^H- and ^13^C-NMR spectra of ILs dissolved in deuterated water (D_2_O) were collected at RT using a Bruker DPX 400 MHz spectrometer (Karlsruhe, Germany). ATR-FTIR spectra of ILs and membranes were recorded on a Bruker Alpha-II with a Diamond ATR accessory from Bruker Optics GmbH (Billerica, MA, USA) at RT. Thermogravimetric analysis (TGA) of ILs and membranes was performed with a Q500 analyzer (TA Instruments, New Castle, DE, USA) under N_2_ flow (50 cm^3^ min^−1^) from 25 to 800 °C with a heating rate of 10 °C min^−1^. Differential scanning calorimetry (DSC) of samples was carried out with a PerkinElmer DSC Q100 instrument (TA Instruments, New Castle, DE, USA) under N_2_ flow from −90 to 100 °C at 10 °C min^−1^. The morphology and cross-section of the polymeric composite membranes were determined by scanning electron microscopy (SEM) using an LEO Supra 35VP microscope (Zeiss, Oberkochen, Germany). Finally, surface wettability was evaluated by contact angle measurements using a surface tensiometer (OCA-40, Dataphysics, Filderstadt, Germany) and the sessile drop method.

### 2.5. Membrane Permeation Properties

Membrane permeation properties of PLA and IL/PLA composites were studied using the Wicke–Kallenbach method at 30 °C. Details of the experimental setup and the permeation cell have already been described in earlier studies [15,16]. Regarding single-gas experiments, a feed flow rate of 25 cm^3^ min^−1^ of each gas (CO_2_, CH_4_, H_2_, N_2_) and a He sweep flow rate of 20 cm^3^ min^−1^ were used at 1 atm. In the case of PLA and 3%[Ch][Gly]/PLA, a dry gas mixture of H_2_:CO_2_ (3:1) was used, representing the product stream after methane reforming. Further study was conducted, using the same mixture under humid conditions (4 wt.% H_2_O), where the mixture flow was 50 cm^3^ min^−1^ and the He sweep flow was 95 cm^3^ min^−1^.

Permeability (P_i_) of gas component i and the selectivity or separation factor (α_ij_) for a binary gas pair i and j were calculated using Equations (5) and (6):

(5) $$P_{i}=\frac{y_{i}\times \dot{V}}{A}\times \frac{l}{\Delta P}$$

(6) $$\alpha_{ij}=\frac{P_{i}}{P_{j}}$$

where y_i_ is the volumetric fraction of component i in permeate side, $\dot{V}$ is the sweep gas volumetric flow rate in cm^3^ min^−1^, A equals 6.97 cm^2^, l is the thickness of membrane in cm, and ΔP is the partial pressure difference in cmHg.

### 2.6. Single-Gas Sorption Measurements

CO_2_ sorption measurements were performed at RT and an equilibrium pressure of 1 bar using the variable-pressure technique in a homemade constant-volume apparatus. The solubility coefficient (S) was determined directly from the sorption experiments, while the diffusion coefficient (D) was subsequently calculated using the solution–diffusion model from the experimentally measured permeability (P) and solubility (S) coefficients according to Equation (7):

(7) $$P_{i}=S_{i}\times D_{i}$$

## 3. Results and Discussion

### 3.1. Preparation and Characterization of IL/PLA Composite Membranes

**Figure 1:**
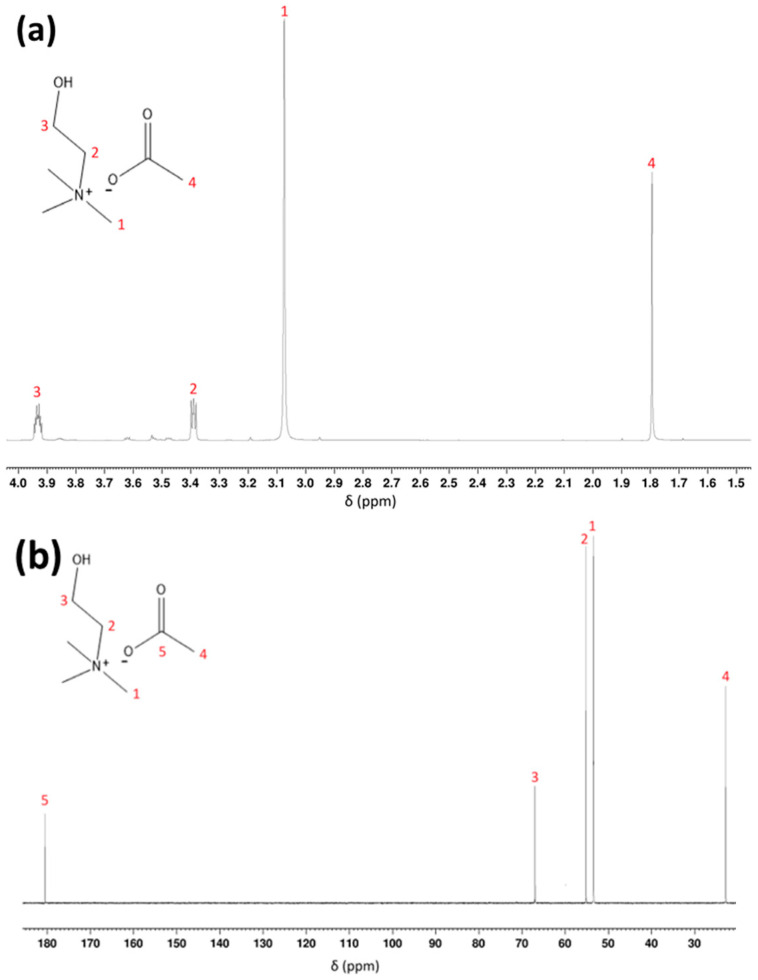
(**a**) ^1^H-NMR (top) and (**b**) ^13^C-NMR (bottom) spectra of [Ch][Gly] in D_2_O.

Initially, three cholinium ionic liquids (ILs) containing acetate, glycinate, and lysinate anions were synthesized via an acid–base reaction. Their structures were confirmed by ^1^H and ^13^C NMR spectroscopy. Figure 1 presents the spectra of [Ch][Ac], while the spectra of the remaining ILs are provided in the Supplementary Information (Figures S1 and S2). The structure of ILs was also confirmed via ATR-FTIR spectroscopy (Figure S3, Supporting Information). The bands attributed to the COO^−^ group are evident. Specifically, the asymmetric stretching of C=O peak at 1566 cm^−1^ and the vibration of C-O at 1057 cm^−1^ are confirmed [17].

**Figure 2:**
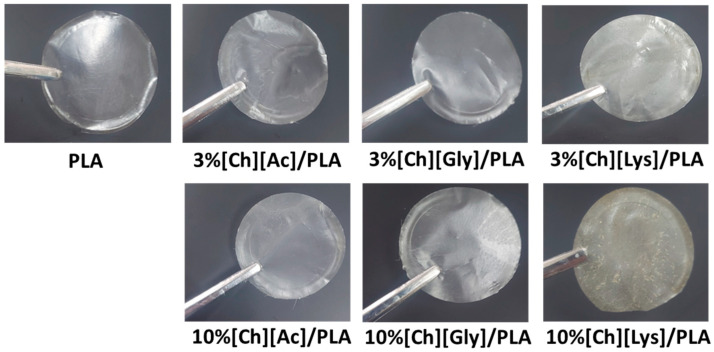
Pictures of the prepared neat PLA and IL/PLA composite membranes.

Subsequently, the incorporation of ILs into the PLA matrix yielded flexible, soft membranes (see Figure 2), in contrast to the rigid and brittle nature of neat PLA. It is worth noting that PLA composite membranes with 10 wt.% [Ch][Ac] and 10 wt.% [Ch][Lys] showed limited transparency. One possible explanation is that the use of acetone during casting method induces the precipitation of ILs, resulting in aggregate formation. The addition of ILs into the PLA matrix was verified via ATR-FTIR spectroscopy (Figure S4, Supporting Information). The asymmetric stretching of C=O band at 1566 cm^−1^ is evident for all composite membranes.

**Figure 3:**
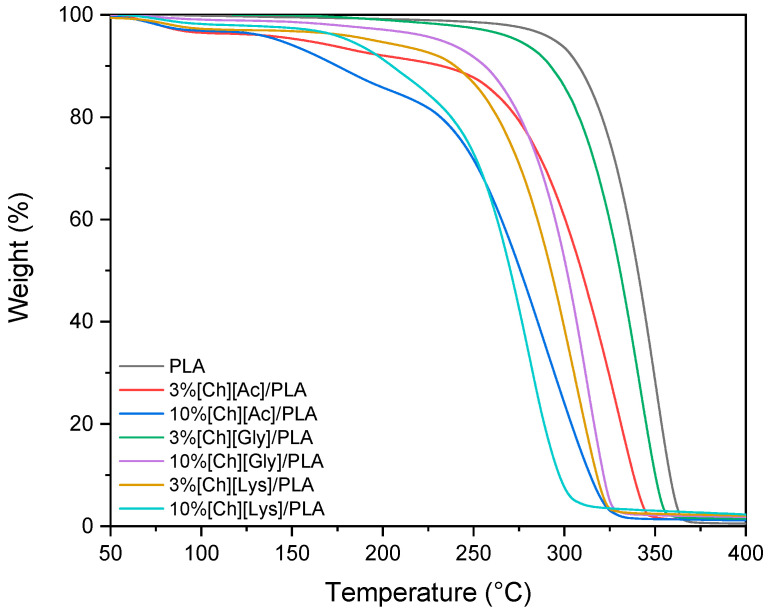
Comparative TGA curves of PLA and IL/PLA composite membranes.

The thermal stability of IL/PLA composite membranes was investigated through thermogravimetric analysis (TGA). As illustrated in Figure 3, neat PLA shows one degradation step with its maximum degradation temperature at 350 °C [18]. Also, the degradation of PLA starts at 295 °C (T_d,5%_) and it is completed at 400 °C. Regarding composite membranes containing [Ch][Ac] IL, three degradation steps are observed. An initial 3 wt.% weight loss up to 130 °C can be attributed to moisture removal, while the second weight loss between 130 °C and 220 °C or 240 °C (3 wt.% and 10 wt.%, respectively) is due to the degradation of the ammonium cation of neat [Ch][Ac] IL (see also Figure S5, Supporting Information) [19]. The higher-temperature degradation step is assigned to PLA. Notably, increasing the IL loading results in a systematic reduction in the PLA degradation temperature, shifting from 350 °C in neat PLA to 330 °C and 290 °C for 3%[Ch][Ac]/PLA and 10%[Ch][Ac]/PLA, respectively. The presence of acetate anions is anticipated to catalyze hydrolysis and/or thermal oxidation processes, resulting in increased ester bond cleavage and, consequently, enhanced degradation of PLA. In the case of [Ch][Gly]-based composite membranes, only one degradation step can be noted with maximum degradation temperature at 340 °C and 312 °C for 3%[Ch][Gly]/PLA and 10% [Ch][Gly]/PLA, respectively. The absence of a distinct degradation step corresponding to the IL may be indicative of good miscibility with PLA, suggesting the formation of a homogeneous system. Also, the thermal stability of the 3% [Ch][Gly]/PLA composite membrane shows minimal deviation from that of neat PLA, highlighting its potential suitability for gas separation applications. Considering membranes with [Ch][Lys], a similar trend to that observed in [Ch][Ac]-based membranes is evident. Notably, these membranes exhibit the lowest thermal stability, with maximum degradation temperatures of 305 °C and 280 °C, which are substantially lower than those of the respective membranes with equivalent IL content.

**Figure 4:**
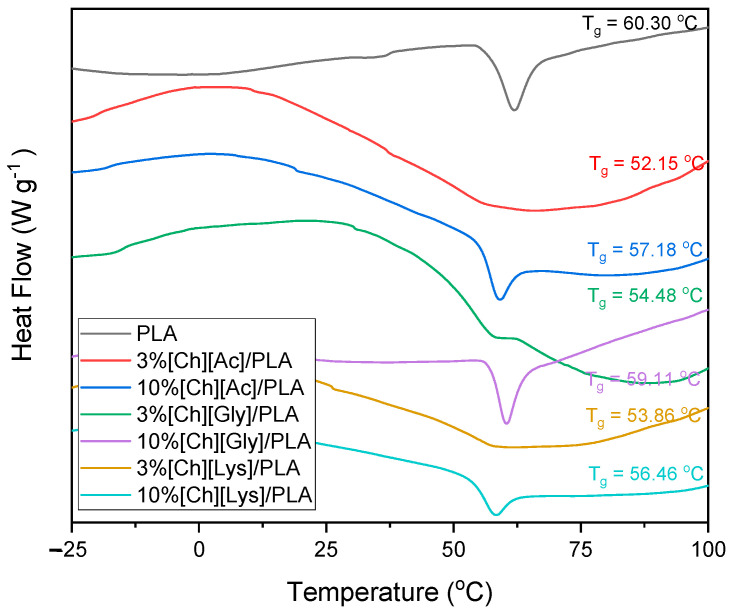
DSC curves of PLA and IL/PLA composite membranes.

The thermal properties of membranes were also studied using differential scanning calorimetry (DSC), thus determining their glass transition temperature (T_g_). Generally, the addition of IL in a polymer matrix leads to lower T_g_ when ILs act as plasticizers [20]. Plasticizers weaken intermolecular interactions between polymer chains within the matrix, thereby increasing the intrinsic free volume and reducing the energy barrier for segmental motion. As a result, polymer segments require less thermal energy to undergo cooperative rearrangements, leading to a decrease in the T_g_. This effect is consistently observed upon incorporating 3 wt.% of any IL into the PLA matrix, with T_g_ decreasing approximately 6 to 8 °C compared to the T_g_ of neat PLA (Figure 4). On the other hand, the T_g_ of composite membranes with the higher IL content seem to return close to the initial T_g_ value of precursor polymer. This suggests poor miscibility of ILs with PLA and limited plasticization of the membranes. This finding is consistent with the reduced transparency discussed earlier. Regarding the influence of the anion’s chemical structure on T_g_, it is also observed that, at lower IL concentrations, T_g_ remains essentially unchanged within experimental errors. This indicates that the anion molar volume does not play a significant role under these conditions.

**Figure 5:**
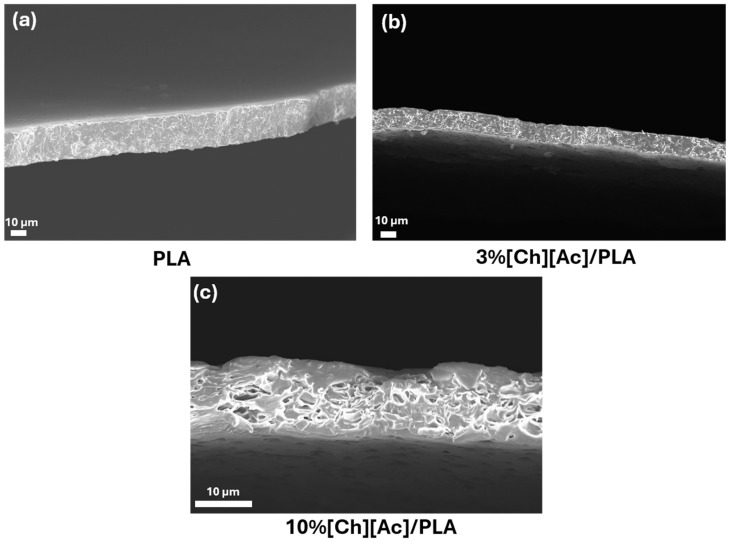
SEM micrographs of (**a**) PLA membrane, (**b**) 3%[Ch][Ac]/PLA and (**c**) 10%[Ch][Ac]/PLA composite membranes.

The cross-sectional SEM micrographs of the fabricated membranes are presented in Figure 5 and Figure S6 of the Supporting Information. The images reveal that both neat PLA and composite membranes containing 3 wt.% IL exhibit compact, homogeneous, and non-phase-separated morphologies. This observation, in agreement with the DSC analysis, suggests that ILs are uniformly dispersed and well-integrated within the polymer matrix. In contrast, composite membranes with higher IL loadings—particularly 10% [Ch][Ac]/PLA and 10% [Ch][Lys]/PLA—display more porous, irregular, and heterogeneous structures. This behavior can be attributed to the limited compatibility and poorer miscibility between the ILs and PLA at elevated concentrations, as also supported by the DSC results.

Complementary information from the surface morphology (Figure S7) further supports these observations. The surface of neat PLA exhibits noticeable roughness, characterized by micro-scale peaks and valleys. Upon the incorporation of 3 wt.% IL, a clear reduction in surface roughness is observed. Among all compositions, the membrane containing 3 wt.% [Ch][Gly] shows the most favorable morphology, with a very smooth, dense, and defect-free surface. However, when the IL content is increased to 10 wt.%, a slight increase in surface roughness is observed. On the other hand, 10%[Ch][Ac]/PLA and 10% [Ch][Lys]/PLA membranes exhibit pronounced agglomeration rather than uniform morphology, leading to significantly higher surface roughness.

### 3.2. Single- and Mixed-Gas Permeation Properties of IL/PLA Composite Membranes

The single-gas permeation properties of PLA and IL/PLA composite membranes are summarized in Table 1. In addition to H_2_ and CO_2_, CH_4_ and N_2_ permeation measurements were also performed to provide a broader understanding of the transport behavior of the prepared membranes. Due to their relatively inert nature and weaker interactions with the membrane matrix, these gases serve as useful reference probes for evaluating the influence of IL incorporation on gas permeability. In general, PLA exhibits relatively low gas permeability [21,22]. Reported CO_2_ permeability values for PLA typically range between 1 and 10 Barrer, while CH_4_ and N_2_ permeabilities are generally found between 0.05 and 1 Barrer. The results obtained in the present study are consistent with these literature values, confirming the inherently low gas transport characteristics of neat PLA membrane. The pure-gas permeabilities follow the order H_2_ (2.87 Å) > CO_2_ (3.30 Å) > CH_4_ (3.80 Å) [23]. This trend indicates that gas diffusion plays a more dominant role in controlling membrane permeability than gas solubility, a pattern that is evident in glassy polymers such as PLA (T_g_ ≈ 60 °C) [23]. In these materials, the rigid polymer matrix restricts molecular motion, making size-selective diffusion the governing transport mechanism. As a result, gases with smaller kinetic diameters permeate more readily through the membrane structure, leading to higher permeability values. Consequently, all H_2_/gas ideal selectivity values are greater than one, reflecting the preferential permeation of H_2_ over the other gases due to its smaller kinetic diameter and faster diffusion through the membrane matrix.

Generally, the incorporation of 3 wt.% ILs into the polymer matrix leads to an increase in gas permeability, as ILs act as plasticizers, increasing the free volume within the membrane structure [24]. This, along with the smoother surface morphology, which contributes to a more controlled transport pathway, results in the enhancement of gas diffusivity, leading to higher overall permeability values. In the case of 10%[Ch][Ac]/PLA and 10%[Ch][Lys]/PLA membranes, DSC and SEM analyses indicate poor miscibility between the ILs and the polymer matrix. This incompatibility resulted in porous structures with pronounced heterogeneities and high roughness. Therefore, their gas transport properties cannot be accurately or reproducibly determined under the present experimental conditions. For this reason, they were excluded from quantitative permeation analysis, and dashed entries are reported in Table 1 for gas permeability and selectivity values. On the other hand, although gas permeabilities could be measured for the 10%[Ch][Gly]/PLA membrane, its gas separation performance was inferior compared to membranes containing lower IL loadings. This suggests that while the incorporation of ILs can enhance gas permeability relative to neat PLA, excessive IL content may adversely affect membrane separation efficiency. Therefore, only a limited amount of IL appears necessary to achieve improved gas transport properties.

Table S1 of the Supporting Information summarizes the CO_2_ solubility and diffusion coefficients obtained for the prepared membranes. The determination of H_2_ solubility coefficient was not feasible using the current experimental setup, as the sensitivity of the apparatus is insufficient to accurately detect the very low sorption levels of this gas.

It can be observed that the incorporation of ILs into the PLA matrix leads to an increase in CO_2_ solubility, as expected given the intrinsic CO_2_-philicity of ILs. However, the enhancement in CO_2_ diffusivity is significantly more pronounced than the corresponding increase in solubility. Specifically, the addition of 3 wt.% [Ch][Gly] results in a threefold increase in the diffusion coefficient compared to neat PLA, whereas the solubility coefficient increases only by a factor of 1.3. Consequently, gas transport through the polymer matrix is markedly enhanced, which can be attributed to an increase in free volume within PLA upon IL incorporation. This interpretation is further supported by the observed decrease in T_g_ from DSC measurements.

With respect to the type of IL used and the constant loading (3 wt.%) employed, it is worth noting that no significant difference is observed between the acetate- and glycinate-based ILs in terms of their effect on permeability. In contrast, for the lysinate-based IL, the increase in permeability—particularly for H_2_—is less pronounced. Specifically, H_2_ permeability is approximately three times lower than that of 3 wt.% [Ch][Gly].

**Figure 6:**
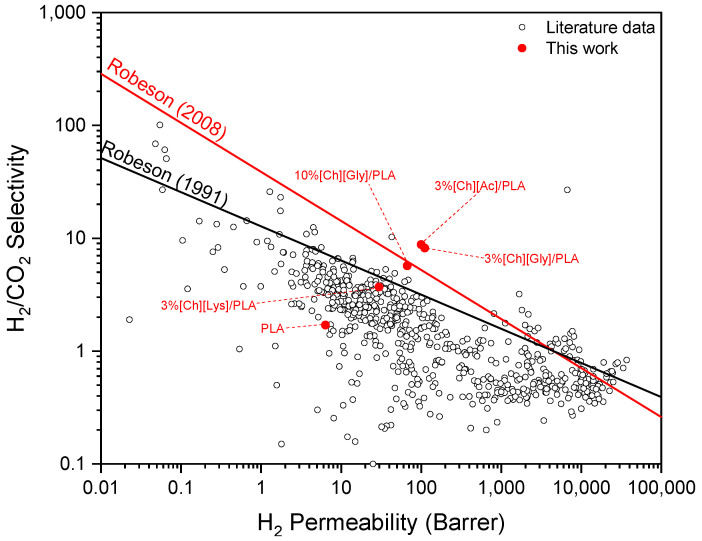
H_2_/CO_2_ selectivity as a function of H_2_ permeability of PLA and IL/PLA composite membranes (red circles). The 1991 and 2008 upper bounds for H_2_/CO_2_ were adapted from refs. [9,27]. The rest of the data points were taken from ref. [28].

The membrane containing 3 wt.% [Ch][Gly] exhibits the highest H_2_ permeability (111 Barrer). Although CO_2_ permeability also increases, the H_2_/CO_2_ selectivity is 8.2. This behavior can be attributed to the fact that gas transport in these systems is predominantly governed by size-sieving effects rather than solubility. As a result, the diffusivity of H_2_ is expected to increase more significantly than the permeability of CO_2_. Consequently, an enhancement in H_2_/CO_2_ selectivity is observed under plasticization conditions. As shown in Figure 6, this performance surpasses the Robeson Upper Bound (2008) when compared with other polymers [8]. In contrast, commercial polymeric membranes, including polyimide (PI), polysulfone (PSf), poly(phenylene oxide) (PPO), and polyetherimide (PEI), generally show more limited H_2_/CO_2_ separation performance, with reported selectivities as low as ~5 (e.g., PEI under mixed-gas conditions). This exceptional combination of permeability and selectivity suggests that the membrane is a promising candidate for H_2_ separation processes following reforming operations. The comparison of gas permeability and the selectivity of PLA and the prepared composite membranes for the H_2_/CH_4_ and H_2_/N_2_ gas pair are also shown in Figures S8 and S9 of Supporting Information. All permeation performance values remain below the Pinnau Upper Bound (2015) [25]. The observed decrease in H_2_/CH_4_ or H_2_/N_2_ selectivity compared to PLA suggests that these membranes may have limited suitability for applications requiring high separation between H_2_ and CH_4_ or N_2_, such as CH_4_ pyrolysis, natural gas upgrading, or H_2_ separation from N_2_ in ammonia (NH_3_) synthesis processes. For this reason, 3% [Ch][Gly]/PLA composite membrane compared to neat PLA was further studied under mixed-gas conditions to simulate the gas stream after reforming processes. PLA membranes containing 1 wt.% and 5 wt.% [Ch][Gly] were also evaluated for CO_2_ and H_2_ permeation (Table S2 of Supporting Information). The 1 wt.% [Ch][Gly]/PLA membrane exhibited gas transport properties comparable to neat PLA, indicating a negligible influence of the IL at this loading. Increasing the [Ch][Gly] content to 5 wt.% led to intermediate permeation behavior between the 1 wt.% and 3 wt.% membranes. Among all compositions investigated, the 3 wt.% [Ch][Gly]/PLA membrane demonstrated the best H_2_/CO_2_ separation performance, confirming it as the optimal IL loading for this membrane system.

A dry H_2_/CO_2_ gas mixture with a molar ratio of 3:1 was employed. As shown in Table 2, the gas permeabilities of neat PLA and 3%[Ch][Gly]/PLA or 10%[Ch][Gly]/PLA composite membranes do not differ significantly from those obtained in single-gas experiments within experimental error, indicating that competitive transport effects are minimal under these conditions. Under humid operating conditions, H_2_ permeability decreases in both membranes, indicating that the presence of water inhibits H_2_ transport. This effect is likely associated with water molecules occupying free volume within the membrane matrix and interfering with the diffusion pathways available for H_2_ permeation. This behavior is also observed in other polymers, such as PVA [26].

In the case of the composite membranes, CO_2_ permeability exhibits a twofold increase under humid conditions. Consequently, the H_2_/CO_2_ separation factor decreases, as the enhanced CO_2_ transport offsets the reduced H_2_ permeability. Although membrane hydrophilicity appears to increase, as indicated by changes in water contact angle, this behavior cannot be attributed to membrane swelling. This conclusion is supported by SEM measurements showing no significant change in membrane thickness before and after humid exposure. The results show that the membrane thickness remains essentially unchanged, within the experimental error (<2%). Instead, the observed increase in CO_2_ permeation points toward the activation of a facilitated transport mechanism. This is consistent with the chemical structure of the incorporated IL, where carboxylate functional groups can act as reversible carriers for carbonate or bicarbonate species formed in the presence of water [29,30]. Such interactions promote selective CO_2_ transport through the membrane, thereby explaining the significant enhancement in permeability under humidified gas streams. Regarding 10%[Ch][Gly]/PLA, it exhibits essentially no separation capability for H_2_/CO_2_ gas pair. In conclusion, the 3%[Ch][Gly]/PLA membrane could potentially be used in H_2_ purification from CO_2,_ but effective H_2_O removal would be necessary prior to membrane application. From an economic perspective, the estimated production cost of the membrane remains highly competitive, ranging between approximately 1.5 and 2.5 USD kg^−1^ [31,32]. This cost is considerably lower than that of inorganic membranes, such as α-alumina (3.25–4.35 USD kg^−1^) [33], while being comparable to conventional polymeric membrane materials, highlighting the potential of the developed membrane as a cost-effective and sustainable alternative for gas separation applications.

## 4. Conclusions

The present study demonstrates that the incorporation of cholinium-based ILs into a PLA matrix represents an effective strategy for tailoring membrane properties toward gas separation applications. At low IL loading (3 wt.%), the membranes exhibited homogeneous morphology, good dispersion of the IL phase, and reduced T_g_s, indicating effective plasticization and increased free volume within the polymer matrix. Among the prepared membranes, the 3%[Ch][Gly]/PLA composite membrane achieved the highest H_2_ permeability while maintaining a competitive H_2_/CO_2_ selectivity, surpassing the Robeson Upper Bound (2008) for this gas pair. Mixed-gas permeation experiments further confirmed the robustness of its separation performance under dry operating conditions, with negligible competitive transport effects relative to single-gas measurements. Under humidified conditions, however, enhanced CO_2_ permeability was observed due to a facilitated transport mechanism, leading to a reduction in H_2_/CO_2_ separation factor. Overall, these findings establish glycinate-based IL/PLA membrane as promising candidate for H_2_ purification from CO_2_-containing streams, particularly in dry post-reforming environments where effective H_2_O management can be ensured.

## Figures and Tables

**Table 1:** Single-gas permeability and H_2_ selectivity over other gases of PLA and composite membranes (1 Barrer = 10^−10^ cm^3^_(STP)_ cm cm^−2^ s ^−1^ cmHg^−1^).

x%IL/PLA Membranes	Thickness (μm)	H_2_ Permeability (Barrer)	CO_2_ Permeability (Barrer)	H_2_/CO_2_ Selectivity	CH_4_ Permeability (Barrer)	H_2_/CH_4_ Selectivity	N_2_ Permeability (Barrer)	H_2_/N_2_ Selectivity
PLA	28	6.4 ± 0.1	3.7 ± 0.7	1.7 ± 0.5	0.142 ± 0.003	54 ± 2	0.11 ± 0.04	68 ± 30
3%[Ch][Ac]	15	100 ± 2	11.3 ± 0.2	8.8 ± 0.3	11.8 ± 0.3	8.5 ± 0.3	7.6 ± 0.5	13.2 ± 0.3
10%[Ch][Ac]	15	-	-	-	-	-	-	-
3%[Ch][Gly]	19	111 ± 3	13.6 ± 0.2	8.2 ± 0.2	7.6 ± 0.2	14.7 ± 0.4	8.2 ± 0.5	13.66 ± 0.08
10%[Ch][Gly]	13	67 ± 2	11.8 ± 0.2	5.7 ± 0.1	11.0 ± 0.3	6.1 ± 0.1	6.8 ± 0.5	9.88 ± 0.08
3%[Ch][Lys]	21	29.8 ± 0.7	8.1 ± 0.1	3.7 ± 0.1	9 ± 1	3.5 ± 0.5	5.14 ± 0.05	5.8 ± 0.2
10%[Ch][Lys]	41	-	-	-	-	-	-	-

**Table 2:** Gas permeation and physical properties of PLA and composite membranes under dry and wet conditions (1 Barrer = 10^−10^ cm^3^_(STP)_ cm cm^−2^ s ^−1^ cmHg^−1^).

x%IL/PLA Membranes	Thickness (μm)	Water Contact Angle (Degrees)	H_2_ Permeability (Barrer)	CO_2_ Permeability (Barrer)	H_2_/CO_2_ Separation Factor
**Single gases**					
PLA	28	79.9 ± 0.5	6.4 ± 0.1	3.7 ± 0.7	1.7 ± 0.5
3%[Ch][Gly]	19	78.3 ± 0.5	111 ± 3	13.6 ± 0.2	8.2 ± 0.2
10%[Ch][Gly]	13	58.1 ± 0.5	67 ± 2	11.8 ± 0.2	5.7 ± 0.1
**Dry conditions**					
PLA	28	79.9 ± 0.5	5.1 ± 0.1	4.3 ± 0.8	1.2 ± 0.3
3%[Ch][Gly]	19	78.3 ± 0.5	118 ± 3	13.7 ± 0.2	8.6 ± 0.2
10%[Ch][Gly]	13	58.1 ± 0.5	65 ± 2	11.6 ± 0.2	5.6 ± 0.1
**Wet conditions**					
PLA	28	79.9 ± 0.5	<0.89 ± 0.02	4.2 ± 0.1	>0.212 ± 0.008
3%[Ch][Gly]	19	78.3 ± 0.5	46 ± 3	26.1 ± 0.2	1.8 ± 0.1
10%[Ch][Gly]	13	58.1 ± 0.5	24 ± 3	23.4 ± 0.2	1.03 ± 0.02

## Data Availability

The original contributions presented in this study are included in the article/Supplementary Material. Further inquiries can be directed to the corresponding author.

## Supplementary Materials

The following supporting information can be downloaded at https://www.mdpi.com/article/10.3390/ma19122567/s1. Figure S1. ^1^H-NMR (left) and ^13^C-NMR (right) spectra of [Ch][Gly] in D_2_O; Figure S2. ^1^H-NMR (left) and ^13^C-NMR (right) spectra of [Ch][Lys] in D_2_O; Figure S3. ATR-FTIR spectra of synthesized cholinium ILs with acetate, glycinate and lysinate anions; Figure S4. ATR-FTIR spectra of the prepared neat PLA and IL/PLA composite membranes; Figure S5. TGA curves of the prepared ILs; Figure S6. Cross-sectional images of (a) 3%[Ch][Gly]/PLA, (b) 10%[Ch][Gly]/PLA, (c) 3%[Ch][Lys]/PLA and (d) 10%[Ch][Lys]/PLA composite membranes; Figure S7. Surface SEM micrographs of (a) PLA, (b) 3%[Ch][Ac]/PLA, (c) 10%[Ch][Gly]/PLA, (d) 3%[Ch][Gly]/PLA, (e) 10%[Ch][Gly]/PLA, (f) 3%[Ch][Lys]/PLA and (g) 10%[Ch][Lys]/PLA composite membranes; Figure S8. H_2_/CH_4_ selectivity as a function of H_2_ permeability of PLA and IL/PLA composite membranes (red circles). The 2008 and 2015 upper bounds for H_2_/CH_4_ were adapted from refs. [9,25]. The rest of the data points were taken from ref. [28]; Figure S9. H_2_/N_2_ selectivity as a function of H_2_ permeability of PLA and IL/PLA composite membranes (red circles). The 2008 and 2015 upper bounds for H_2_/CH_4_ were adapted from refs. [9,25]. The rest of the data points were taken from ref. [28]; Table S1. Pure CO_2_ solubility and diffusivity coefficients and CO_2_ permeability of PLA and composite membranes at 1 bar and 25 °C; Table S2. Single gas permeability and H_2_ selectivity over other gases of PLA and composite membranes (1 Barrer = 10^−10^ cm^3^(STP) cm cm^−2^ s ^−1^ cmHg^−1^).
