# Supplementary material for: Tailoring PLA-Based Composite Membranes with Ionic Liquids for Efficient H2/CO2 Separation in Reforming Processes
Source: Materials (Basel). 2026 Jun 13;19(12):2567. doi: 10.3390/ma19122567 (PMC13303151; doi:10.3390/ma19122567)
Supplement: Supplementary file 1 [file materials-19-02567-s001.zip › materials-4356699-supplementary.pdf]

Supplementary Information

Tailoring PLA-Based Composite Membranes with Ionic Liquids for Efficient H_2/CO_2 Separation in Reforming Processes

Dionysios Vroulias *, Athina Nikolopoulou , Theophilos Ioannides and Vassilios Dracopoulos

Foundation for Research and Technology-Hellas, Institute of Chemical Engineering Sciences (FORTH/ICE-HT), GR-26504 Patras, Greece

FIGURES

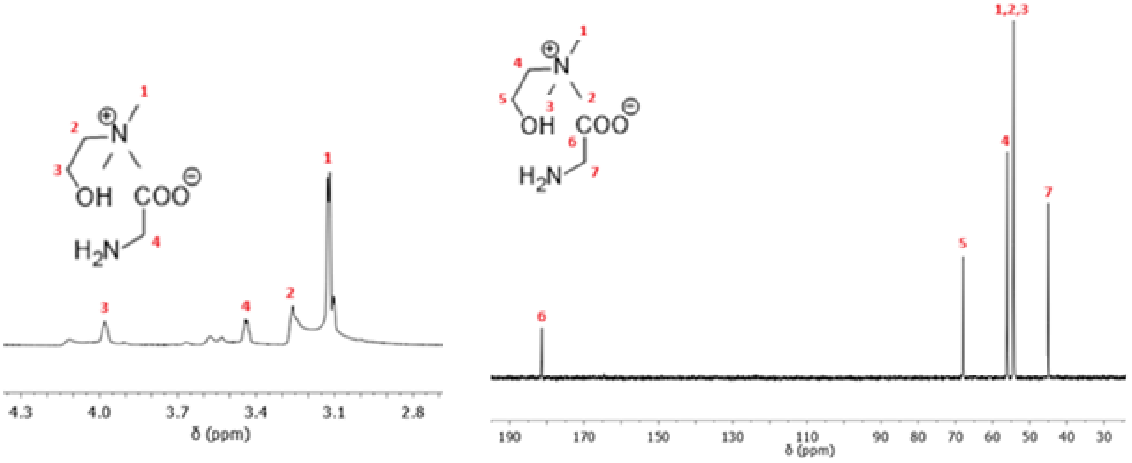

Figure S1. ^{1}H-NMR (left) and ^{13}C-NMR (right) spectra of [Ch][Gly] in D_2O.

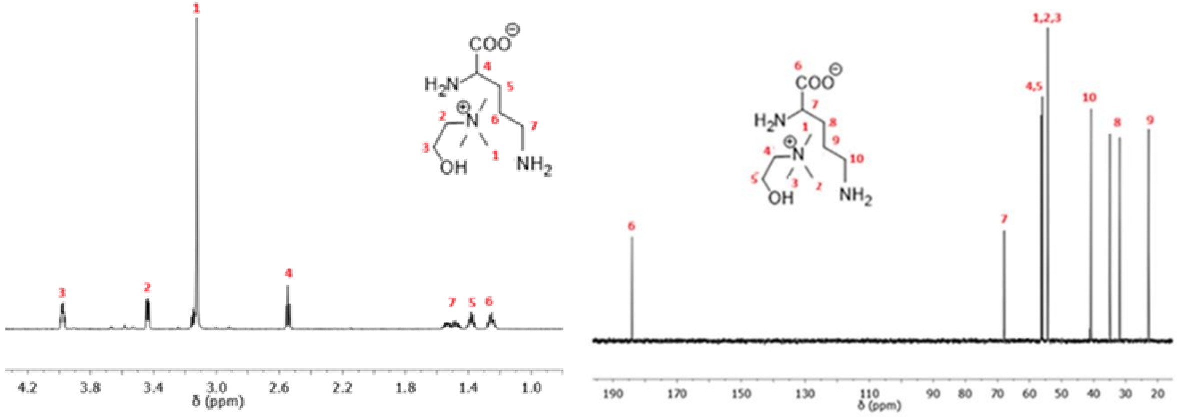

Figure S2. ^{1}H-NMR (left) and ^{13}C-NMR (right) spectra of [Ch][Lys] in D_2O.

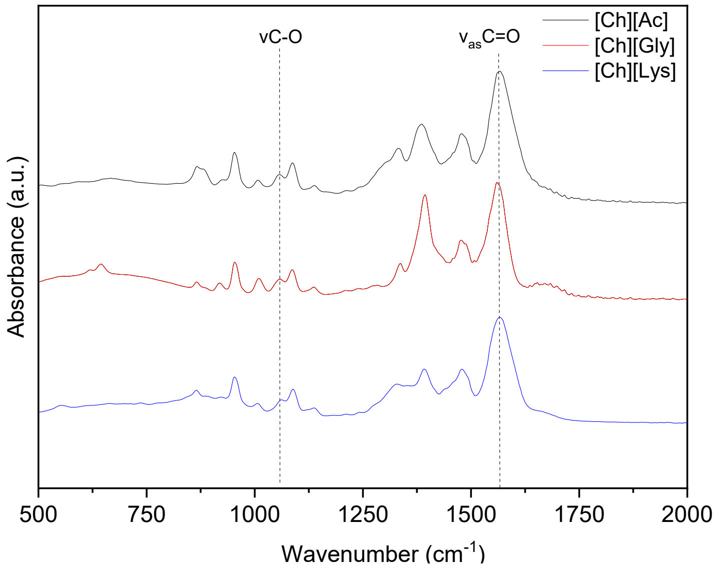

Figure S3. ATR-FTIR spectra of synthesized cholinium ILs with acetate, glycinate and lysinate anions.

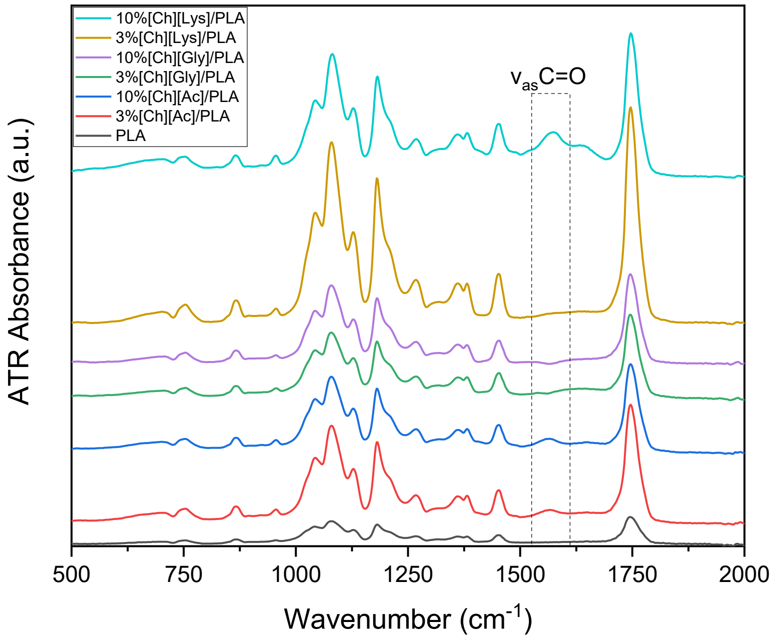

Figure S4. ATR-FTIR spectra of the prepared neat PLA and IL/PLA composite membranes.

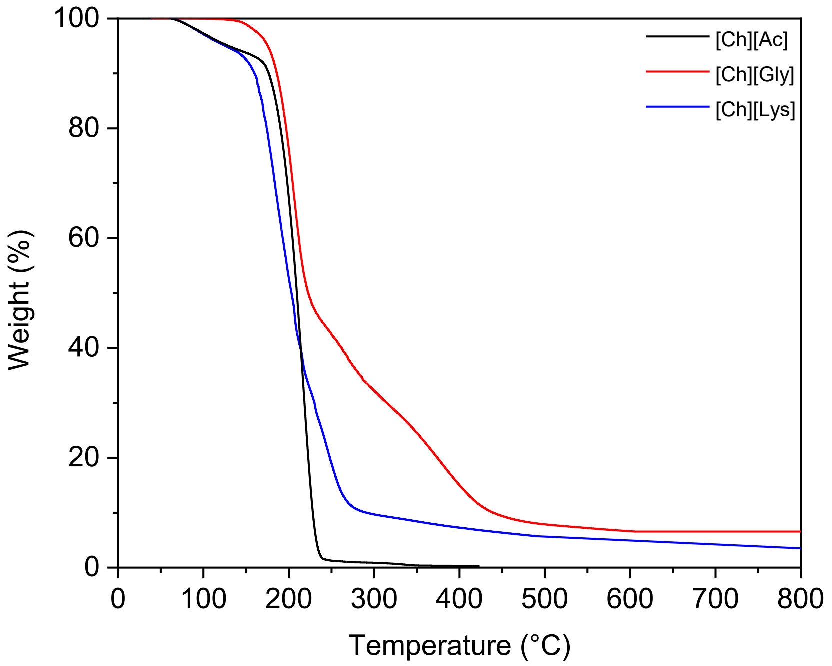

Figure S5. TGA curves of the prepared ILs.

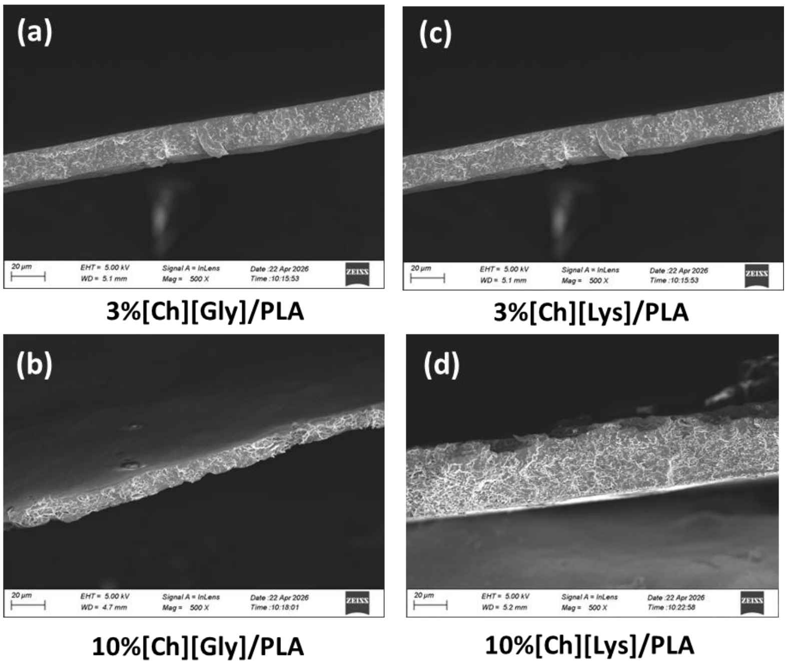

Figure S6. Cross-sectional images of (a) 3%[Ch][Gly]/PLA, (b) 10%[Ch][Gly]/PLA, (c) 3%[Ch][Lys]/PLA and (d) 10%[Ch][Lys]/PLA composite membranes.

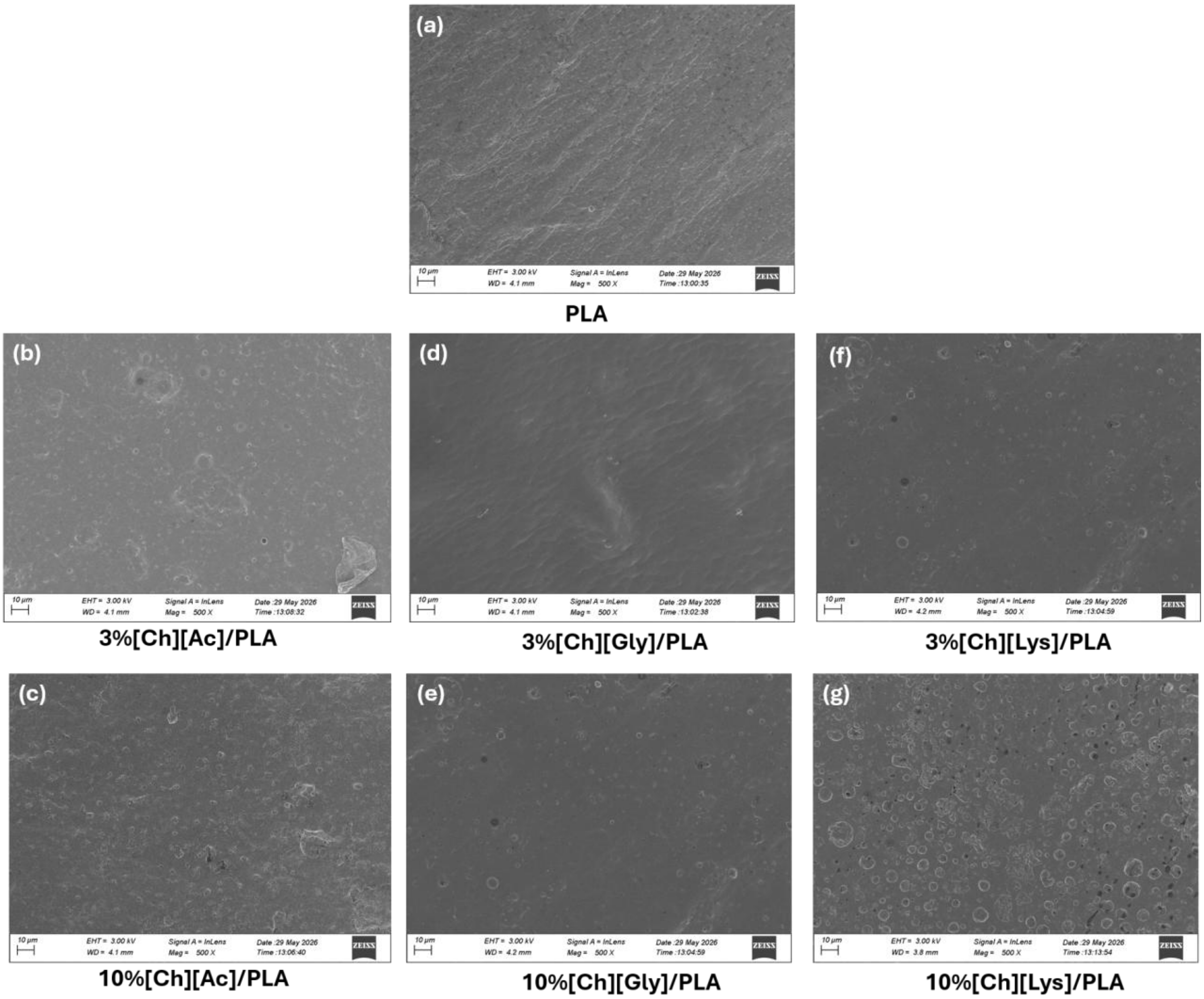

Figure S7. Surface SEM micrographs of (a) PLA, (b) 3%[Ch][Ac]/PLA, (c) 10%[Ch][Gly]/PLA, (d) 3%[Ch][Gly]/PLA, (e) 10%[Ch][Gly]/PLA, (f) 3%[Ch][Lys]/PLA and (g) 10%[Ch][Lys]/PLA composite membranes.

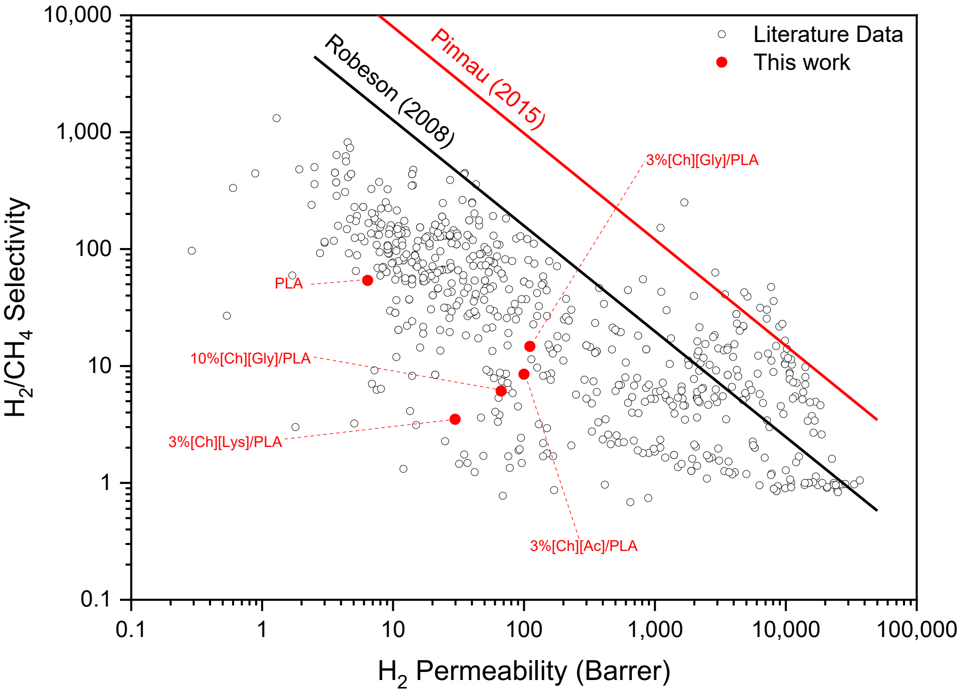

Figure S8. H_2/CH_4 selectivity as a function of H_2 permeability of PLA and IL/PLA composite membranes (red circles). The 2008 and 2015 upper bounds for H_2/CH_4 were adapted from refs. [1, 2]. The rest of the data points were taken from ref. [3].

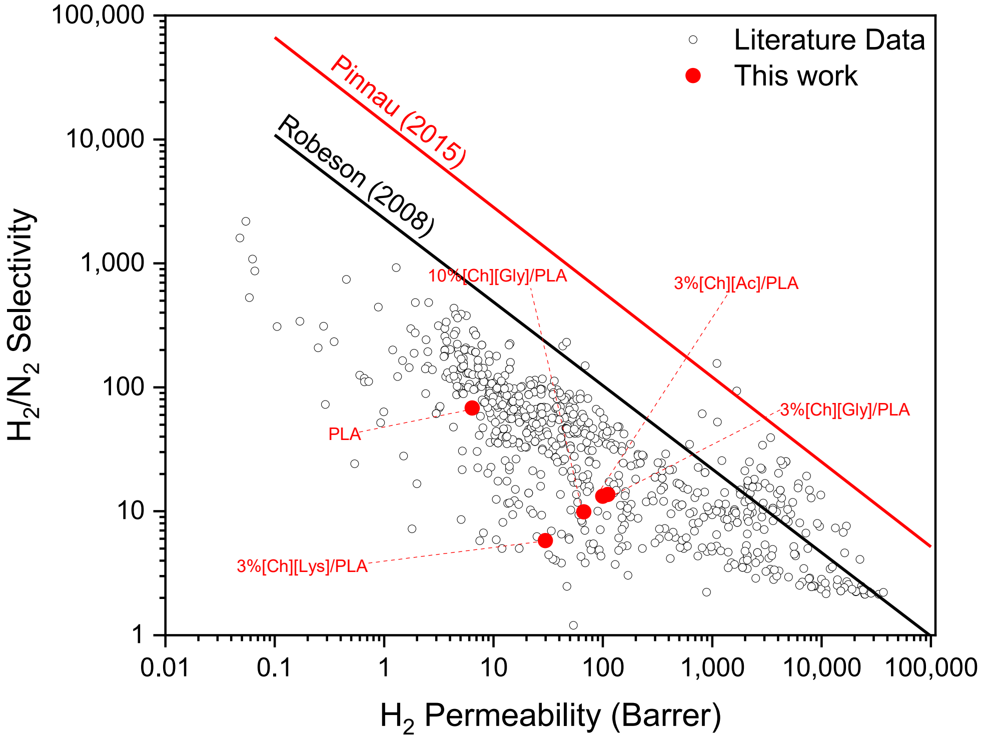

Figure S9. H_2/N_2 selectivity as a function of H_2 permeability of PLA and IL/PLA composite membranes (red circles). The 2008 and 2015 upper bounds for H_2/CH_4 were adapted from refs. [1, 2]. The rest of the data points were taken from ref. [3].

Table S1. Pure CO_2 solubility and diffusivity coefficients and CO_2 permeability of PLA and composite membranes at 1 bar and 25 °C.

x%IL/PLA Membranes	CO_2 Solubility Coefficient (10^{-2} cm^3(STP) cm^{-3} cmHg^{-1})	CO_2 Diffusivity Coefficient (10^{-8} cm^2 s^{-1})	CO_2 Permeability (Barrer)
PLA	4.87	0.76	3.7
3%[Ch][Ac]	12.25	0.92	11.3
3%[Ch][Gly]	6.33	2.14	13.6
10%[Ch][Gly]	6.92	1.71	11.8
3%[Ch][Lys]	7.12	1.14	8.1

Table S2. Single gas permeability and H_2 selectivity over other gases of PLA and composite membranes (1 Barrer = 10^{-10} cm$^3_{(STP)}$ cm cm^{-2} s^{-1} cmHg^{-1}).

x%IL/PLA Membranes	Thickness (μm)	H_2 Permeability (Barrer)	CO_2 Permeability (Barrer)	H_2/CO_2 Selectivity
PLA	28	6.4 ± 0.1	3.7 ± 0.7	1.7 ± 0.5
1%[Ch][Gly]	25	12.1 ± 0.1	4.2 ± 0.3	2.9 ± 0.2
3%[Ch][Gly]	19	111 ± 3	13.6 ± 0.2	8.2 ± 0.2
5%[Ch][Gly]	16	85 ± 2	12.5 ± 0.2	6.8 ± 0.2
10%[Ch][Gly]	13	67 ± 2	11.8 ± 0.2	5.7 ± 0.1

References

1. Robeson, L.M. The upper bound revisited. *J. Membr. Sci.* **2008**, *320(1-2)*, 390–400. doi:10.1016/j.memsci.2008.04.030.
2. Swaidan, R.; Ghanem, B.; Pinnau, I. Fine-Tuned Intrinsically Ultramicroporous Polymers Redefine the Permeability/Selectivity Upper Bounds of Membrane-Based Air and Hydrogen Separations. *ACS Macro Lett.* **2015**, *4(9)*, 947–951. doi:10.1021/acsmacrolett.5b00512.
3. Thornton, A.W.; Freeman; B.D. Robeson, L.M. Polymer Gas Separation Membrane Database (2012). Available online: https://membrane-australasia.org/polymer-gas-separation-membrane-database (accessed on 2 May 2026).
